# Published Research on COVID-19 in the Eastern Mediterranean Region: Bibliometric Analysis

**DOI:** 10.2196/38935

**Published:** 2022-07-19

**Authors:** Randa K Saad, Sara Abu Khudair, Maha El Rabbat, Mayeh Omar, Mohannad Al Nsour, Yousef Khader, Salman Rawaf

**Affiliations:** 1 Global Health Development| Eastern Mediterranean Public Health Network Amman Jordan; 2 Public Health Department Faculty of Medicine Cairo University Cairo Egypt; 3 Nuffield Centre for International Health and Development Leeds Institute of Health Sciences University of Leeds Leeds United Kingdom; 4 Department of Public Health Faculty of Medicine Jordan University of Science and Technology Irbid Jordan; 5 Department of Life Sciences School of Public Health Imperial College London London United Kingdom

**Keywords:** COVID-19, Eastern Mediterranean Region, bibliometric analysis, literature, research, health care system, social inequality, epidemiology, depression, research trend, bibliometry

## Abstract

**Background:**

The challenges presented by the COVID-19 pandemic have led to unprecedented global research activity. The Eastern Mediterranean Region (EMR) continues to contribute to COVID-19 research driven by the unique challenges of the region, including the protracted conflicts, already stressed health systems, and serious health and social inequalities.

**Objective:**

This study aims to provide an overview of the publication activities and trends in COVID-19 research in the EMR from the onset of the disease to early 2022 using bibliometric methods.

**Methods:**

A literature search using Scopus was conducted from December 1, 2019, to January 31, 2022, using keywords relevant to COVID-19 and the World Health Organization (WHO) EMR country list. Data were exported and analyzed using Microsoft Excel and the Citation Overview function on Scopus. The quality of journals was determined using SCImago Journal Rank and CiteScore. VOSviewer software was used to visualize the relationships between authors, countries, and key terms used in the retrieved documents.

**Results:**

A total of 6880 documents were retrieved, of which 1805 (26.24%) were from the Kingdom of Saudi Arabia (KSA) and 1782 (25.90%) from Iran, followed by Pakistan, Egypt, and Jordan. Most published documents were affiliated with EMR universities, primarily the Tehran University of Medical Sciences in Iran and King Saud University in KSA (396/6880, 5.76%, and 370/6880, 5.4%, respectively), while only 407 (5.92%) of 6880 documents were associated with universities outside the EMR. For most of the identified publications (5020/6880, 72.97%), no funding source was reported, while King Saud University contributed the largest share (282/1860, 15.16%) of funded publications. Retrieved documents were cited 53,516 times, with an average of 7.78 (SD 34.30). Iran was the EMR country with the most links to other countries (77 links and total link strength of 1279). The 5 authors with the most publications were from KSA, Qatar, and Jordan. There were 290 high-frequency keywords that occurred ≥10 times and were linked in 7 different clusters. The cluster with the most linked keywords was related to epidemiology and mortality. Recent topics included vaccines, vaccination, machine learning, and online learning.

**Conclusions:**

This is the first study to show trends in and project future developments of COVID-19 research activity in the EMR. Authors and institutions who led research on COVID-19 in the region were from Iran and KSA. There were multiple regional collaborative efforts; however, international collaboration was limited. Recently, interest has been shifting toward topics related to vaccination, machine learning, and online learning. Understanding the current state of research is instrumental to future research production, and our study will inform regional research initiatives on emerging concepts, as well as opportunities for collaboration and funding.

## Introduction

The COVID-19 pandemic continues to be a major global challenge, placing a heavy burden on the economy, social life, public health systems, and the delivery of health services in all countries worldwide [1]. Despite the rapid development of various vaccines to prevent severe illness and treatment modalities, the world still faces numerous challenges in controlling COVID-19 and its impacts [1]. Disparities in global access to vaccines, increasing vaccine hesitancy, impractical long-term implementation of preventive public health measures, and the continuous emergence of new variants are some of these challenges [1-3]. The Eastern Mediterranean Region (EMR) remains at high risk of COVID-19 case surges with the associated short- and long-term consequences primarily due to the combination of the aforementioned causes, added to which are the specific regional environment with protracted conflicts; high denial attitude toward the pandemic; chronically stressed health systems; and the presence of health, economic, and social inequities [4-7].

As of April 1, 2022, there have been 21,576,432 confirmed cases and 340,628 deaths attributable to COVID-19 in the EMR [8]. The EMR has a population of nearly 700 million people in 22 countries with high susceptibility to infectious diseases due to numerous political, economic, social, cultural, and human-animal interactions [5,9]. Almost 40% of the world’s population in need of humanitarian assistance lives in the EMR and is at high risk of coronavirus transmission and associated severe consequences due to overpopulation, suboptimal sanitation, a high caseload of noncommunicable diseases (NCDs), and limited resources and health system capacity [5]. Furthermore, a number of EMR countries have limited testing facilities, weak health system infrastructure and response, and inadequate vital registration and documentation, all of which have been associated with the possibility of underreporting or undertesting or both of COVID-19 cases [4,5,10]. The introduction of vaccination programs in the EMR continues to be hampered by logistical, economic, security, and population hesitancy issues, as well as reaching people living in hard-to-reach areas [11].

COVID-19 had a strong impact on scientific publications worldwide, as evidenced by the rapid increase in the volume and pace of COVID-19 publications, with many journals dedicating special sections or issues to COVID-19, even at the expense of other topics [12,13]. The COVID-19 crisis has also stimulated an unprecedented level of multidisciplinary research involving not only those with a direct interest in COVID-19 research and health, such as virologists, epidemiologists, and clinicians, but also researchers from diverse fields, such as artificial intelligence and business [14,15]. The projected long-term impact of COVID-19 and the fact that it affects multiple areas of life have led to a burst of studies examining the impact of COVID-19 on many nonmedical and nonclinical research areas, such as education, business and management, tourism, and agricultural food supply [16-19]. The global research funding mechanisms and magnitude have also changed. Between the beginning of January 2020 and July 2021, more than US $21.7 trillion was reportedly committed globally to different COVID-19 activities according to data analysis on Devex’s funding platform [20]. Some governments with limited fiscal space or limited prearranged funding sources had to reallocate their existing budgets and allocate funds to COVID-19 studies, even at the expense of other disease control programs, such as for malaria and HIV/AIDS in Africa [21,22]. In the EMR, COVID-19 research has flourished, with countries and organizations dedicating special effort, in addition to technical and financial support for such research [23,24]. Our aim is to provide an overview of the published research activities and trends in COVID-19 research in the EMR by applying bibliometric methods to identify research collaborations, trends, and emerging research themes.

## Methods

### Search Strategy

We conducted a literature search on January 31, 2022, using the Scopus database, covering the period from December 1, 2019, to January 31, 2022. Scopus was chosen for its data mining and bibliometric functions, as well as its compatibility with Visualization of Similarities Viewer (VOSviewer) software. Additionally, Scopus is the largest indexing database, combining the characteristics of both PubMed and Web of Science, and thus allows for enhanced utility, both for literature research and academic needs, including citation analysis [25]. As Scopus does not use subject headings but instead assigns index terms to papers, our search query included the field code KEY, which combines both author keywords and indexed terms [26]. Our search query included keywords relevant to COVID-19 and the World Health Organization (WHO) list of countries in the EMR (Multimedia Appendix 1) [27]. Keywords for COVID-19 included “severe acute respiratory syndrome coronavirus-2,” “SARS-CoV-2,” “sars-2,” and “2019-nCoV,” as well as the words “novel,” “new,” “2019,” “Wuhan,” “Hubei,” and “China” adjacent to the words “coronavirus” and “COVID.” We additionally used wildcards and truncations, as needed, to refine the search. We limited the search to papers, reviews, chapters, and books published in English, associated with any of the EMR countries. We included both published papers and papers in press (accepted for publication by a journal but not assigned to a specific journal issue).

### Scientific Literature Bibliometric Indicators

We exported the data from Scopus to Microsoft Excel and calculated the following bibliometric indicators: number of documents published by publication stage (in press or published), number of open-access documents, number of documents by type of publication (original paper, review, book chapter, book), number of documents by year, number of documents published per country, number of documents published per author, number of documents published per organizational affiliation (publications combined into 5 categories of affiliations: universities in the EMR, universities outside the EMR, ministries of health in the EMR, health centers in the EMR, global health agencies), number of documents per funding source, number of documents per journal, and number of documents published by subject category as indexed by Scopus.

### Citations and Quality Assessment

The Citation Overview function in Scopus enabled us to determine the mean, median, and range of citations of all retrieved documents, as well as identify the topmost cited documents. Additionally, author details were reviewed on Scopus to determine the topmost productive authors’ H-index and citation score.

We assessed the quality of the most productive journals on the topic of COVID-19 in the EMR using the 2021 SCImago Journal Rank (SJR), CiteScore (CS), and the Source-Normalized Impact Per Paper (SNIP). The SJR is based on centrality concepts and data from Scopus, and it limits self-citations and falsely inflated quality ranks, while the CS gives a comprehensive, transparent, and current view of a journal’s impact [28]. SNIP is also based on data from Scopus, and it measures impact by weighing citations based on the total number of citations in a subject field, enabling direct comparison of sources in different subject fields [29]. Using the SCImago ranking website, we identified the subject area of each journal and its corresponding SJR rank divided into 4 equal quartiles, with Q1 comprising the quarter of the journals with the highest values, Q2 the second-highest values, Q3 the third-highest values, and Q4 the lowest values [30].

### Visualization of Similarities

To analyze and visualize relationships among authors, countries, and the key terms used in the retrieved publications, we exported the citation information, bibliographical information, abstracts and keywords, and funding details and included references from Scopus into VOSviewer v.1.6.16 (Centre for Science and Technology Studies, Leiden University) [31].

In constructing the networks and maps, we used a clustering resolution of 1 and a minimum cluster size of 12 to eliminate small clusters, and we illustrated each cluster (eg, group of linked authors, countries, or keywords) using a different color. We scaled the maps based on document weights, unless otherwise specified, so the diameter of each label denotes the number of occurrences of the author, country, or keyword specified by the label, in the documents, and the distance between 2 labels represents the degree to which they are associated [32].

Coauthorship network analysis was performed based on the full counting method. When we used authors as the unit of analysis, we excluded papers authored by >25 authors, and we set both the minimum number of papers published by an author and the minimum number of citations of an author at 5 to identify prominent authors who have published on the topic. When we used countries as the unit of analysis, we excluded papers authored by >25 countries and only included countries with ≥5 published papers. We did not place a restriction on the number of citations during the countries’ coauthorship analysis. We identified the number of coauthorship links countries have with one another and the total strength of the links using the “links” and the “total links strength” attributes provided by VOSviewer.

To identify trending topics relating to COVID-19 in the EMR, we used co-occurrence analysis of author keywords occurring at least 10 times in publications. We excluded the names of countries and regions from the list of keywords to focus on scientific themes, and we also excluded all synonyms of COVID-19, which might obscure the results. We applied normalization based on the association strength to eliminate redundancy in similar keywords that define the same concept. Additionally, we mapped the keyword co-occurrence using overlay visualization to determine the evolution of themes with time.

## Results

### Volume and Type of Publications

Our search retrieved 6880 documents. Most were published documents, and only 586 (8.52%) were still in press. Almost 5439 (79.05%) of 6880 documents were open-access documents, and about 6182 (89.85%) were original papers. Reviews and book chapters constituted only 9.29% (639/6880) and 0.86% (59/6880), respectively. The majority of documents (4630/6880, 67.30%) were published in 2021, whereas 1851 (26.90%) and 39 (0.57%) were published in 2020 and 2019, respectively. Since the beginning of 2022 and until January 31, 2022, 360 (5.23%) documents have been published.

### Publication Distribution by Country

**Figure 1:**
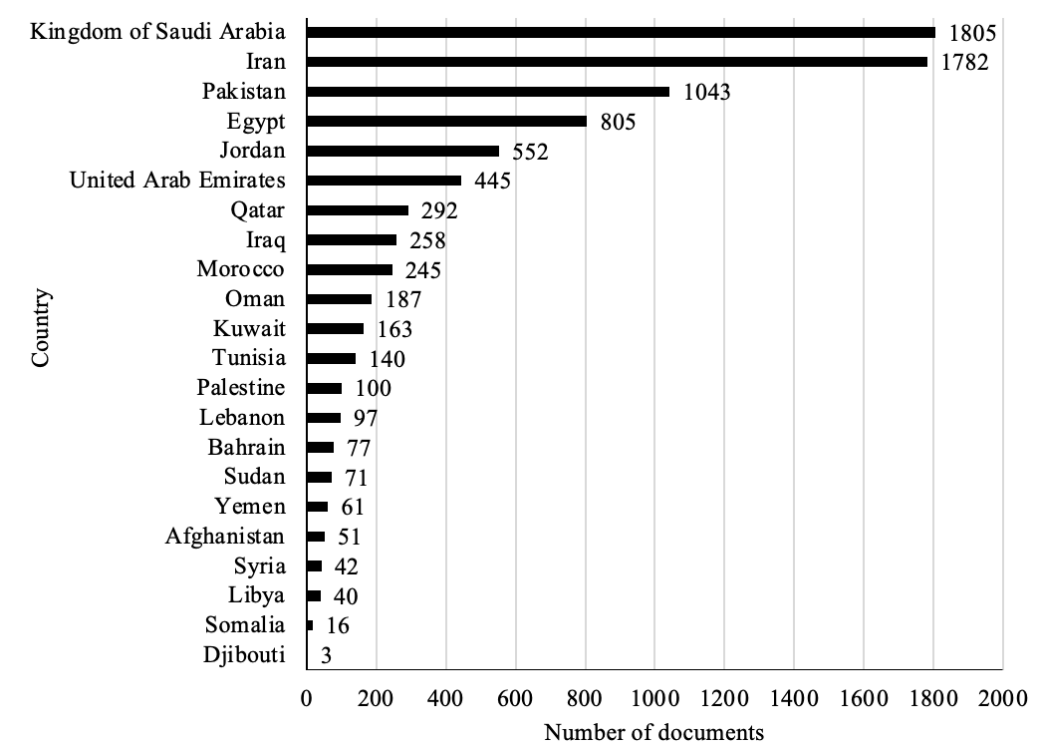
Number of published documents on COVID-19 by country in the EMR. EMR: Eastern Mediterranean Region.

Most publications on COVID-19 in the EMR were from the Kingdom of Saudi Arabia (KSA) and Iran (1805/6880, 26.24%, and 1782/6880, 25.90%, respectively). This was followed by Pakistan (1043/6880, 15.6%), Egypt (805/6880, 11.70%), and Jordan (552/6880, 8.02%). Eight other EMR countries produced ≥100 publications each: the United Arab Emirates (UAE), Qatar, Iraq, Morocco, Oman, Kuwait, Tunisia, and Palestine. However, 5 EMR countries produced <55 publications (ie, Afghanistan, Djibouti, Somalia, Libya, and Syria), where each contributed to <1% (n=3-51) of the overall publication output (Figure 1).

### Publication Distribution by Affiliation

Each publication had 1 or more institutional affiliations. Most of the published documents were associated with 117 universities in the EMR, with 396 (5.76%) and 370 (5.4%) of 6880 documents being associated with the Tehran University of Medical Sciences in Iran and King Saud University in KSA, respectively (Table 1). Of the 6880 identified publications, only 407 (5.92%) were associated with other universities outside the EMR (n=12), most notably the Universiti Sains Malaysia and Johns Hopkins School of Medicine, each associated with 38 (0.55%) of the 6680 documents.

With regard to affiliations with health centers, 10 health centers from the region were associated with publications on COVID-19 in the EMR, half of which were located in KSA (5/10, 50%), namely King Faisal Specialist Hospital and Research Centre, King Abdulaziz Medical City Riyadh, King Fahad Medical City, Johns Hopkins Aramco Healthcare, and King Saud Hospital Riyadh. These 5 health centers, in addition to the Imam Khomeini Hospital in Iran, were each involved with more than 50 publications on the topic.

Ministries of health, including those of KSA, Oman, Kuwait, and Iran, were associated with around 392 (5.69%) of the published 6880 documents, with the Saudi Ministry of Health being the major contributor of 276 (70.41%) of 392 documents. The only international health agency that was associated with COVID-19–related research in the EMR was WHO, being a contributor to 62 (0.90%) of 6880 documents.

**Table 1:** EMR^a^ universities associated with more than 150 published documents on COVID-19 in the EMR.

University	Country	Documents (N=6880), n (%)	Total citations, N	Total citations, excluding self-citations, n (%)	H- index
Tehran University of Medical Sciences	Iran	396 (5.76)	3875	3322 (85.7)	35
King Saud University	KSA^b^	370 (5.38)	2953	2552 (86.4)	26
Shahid Beheshti University of Medical Sciences	Iran	316 (4.59)	2959	2645 (89.4)	27
King Abdulaziz University	KSA	272 (3.95)	2012	1734 (86.2)	23
Iran University of Medical Sciences	Iran	229 (3.33)	2644	2288 (86.5)	25
Cairo University	Egypt	190 (2.76)	2219	2115 (95.3)	14
Jordan University of Science and Technology	Jordan	183 (2.66)	1331	1180 (88.7)	19
Shiraz University of Medical Sciences	Iran	181 (2.63)	2069	1907 (92.2)	23
University of Jordan	Jordan	163 (2.37)	2567	2300 (89.6)	21
Imam Abdulrahman Bin Faisal university	KSA	161 (2.34)	660	582 (88.2)	13
King Saud bin Abdulaziz University for Health Sciences	KSA	157 (2.28)	1169	1046 (89.5)	18

^a^EMR: Eastern Mediterranean Region.

^b^KSA: Kingdom of Saudi Arabia.

### Source of Funding

Most of the identified publications did not report a source of funding (5020/6880, 72.97%). However, of those that did, most were funded by King Saud University (282/1860, 15.16%). The majority of those funded by King Saud University were published in 2022 (183/282, 64.89%). Additionally, Shahid Beheshti University of Medical Sciences in Iran, King Abdulaziz City for Science and Technology in KSA, and King Abdulaziz University in KSA each funded more than 50 publications on the topic (55/1860, 3.00%; 51/1860, 2.74%; and 51/1860, 2.74%, respectively), and most of these funded papers were published in 2022 (98/157, 62.42%).

The US National Institutes of Health (NIH) and the National Natural Science Foundation of China each funded 37 (2%) of 1860 publications. WHO funded 31 (1.67%) of 1860 publications, of which 12 (38.71%) were published in 2020 and 13 (41.94%) in 2021.

### Most Productive Citing Journals

Of the 6821 papers published in journals, 3279 (48.07%) were published in 169 distinct scientific journals, whereas the source of the rest was not defined. The top 15 most productive journals on the topic of COVID-19 in the EMR published 1023 (31.20%) of the 3279 papers (Table 2). Their median 2021 CS and 2021 SJR were 4.5 (range 1.0-18.8) and 0.986 (range 0.283- 2.656), respectively. Of these journals, the *International Journal of Environmental Research and Public Health* (CS=4.5, SJR=0.814) published the maximum papers on COVID-19 in the EMR (155/1023, 15.15%). Most of the top publishing journals were categorized as medical journals or health-related journals, except for 1 journal*, Sustainability Switzerland*, which focuses on energy, environmental science, and social sciences.

**Table 2:** The 2021 CS^a^, SJR^b^, SNIP^c^, H-index, subject area and category, and Scimajo 2020 quarter of the 15 most productive journals on COVID-19 in the EMR^d^.

Journal	Papers (N=3279), n (%)	CS 2021	SJR 2021	SNIP 2021	H-index	Subject area: category; 2021 quarter^e^
*International Journal of Environmental Research and Public Health*	155 (4.72)	4.5	0.814	1.44	138	Environmental science: health, toxicology and mutagenesis, pollution; Q2 Environmental science: health, toxicology, and mutagenesis; Q1 Medicine: public health, environmental and occupational health; Q2
*PLoS ONE*	126 (3.84)	5.6	0.852	1.368	367	Multidisciplinary; Q1
*Frontiers in Public Health*	100 (3.05)	4.0	1.298	1.949	64	Medicine: public health, environmental and occupational health; Q1
*Journal of Infection and Public Health*	85 (2.59)	8.0	1.277	2.018	46	Medicine: infectious diseases; Q1 Medicine: miscellaneous, public health, environmental and occupational health; Q1
*Annals of Medicine and Surgery*	63 (1.92)	1.4	0.373	0.965	30	Medicine: miscellaneous, surgery; Q3
*Vaccines*	58 (1.77)	4.5	1.004	1.167	50	Immunology and microbiology: immunology; Q2 Medicine: pharmacology; Q1 Medicine: infectious diseases; Q2 Pharmacology, toxicology, and pharmaceutics: drug discovery, pharmacology; Q1
*International Journal of Infectious Diseases*	56 (1.71)	10.8	1.433	2.494	104	Medicine: infectious diseases, miscellaneous, microbiology; Q1
*Disaster Medicine and Public Health Preparedness*	54 (1.65)	3.8	0.723	1.011	47	Medicine: public health, environmental and occupational health; Q2
*Environmental Science and Pollution Research*	49 (1.49)	6.6	0.831	1.154	132	Environmental science: environmental chemistry and pollution; Q2 Environmental science: health, toxicology, and mutagenesis; Q1 Medicine: miscellaneous; Q2
*Pan African Medical Journal*	48 (1.45)	1.0	0.283	0.509	36	Medicine: miscellaneous; Q3
*BMC Public Health*	47 (1.43)	4.9	1.156	1.703	159	Medicine: public health, environmental and occupational health; Q1
*Risk Management and Healthcare Policy*	46 (1.40)	2.1	0.556	1.007	29	Medicine: health policy, public health, environmental and occupational health; Q2
*Sustainability Switzerland*	46 (1.40)	5.0	0.664	1.31	109	Energy: energy engineering and power technology, renewable energy, sustainability, and the environment; Q2 Environmental science: miscellaneous, management, monitoring, policy and law; Q2 Social sciences: geography, planning and development; Q1
*Frontiers in Psychology*	45 (1.37)	4.0	0.873	1.605	133	Psychology: miscellaneous; Q1
*Journal of Medical Virology*	45 (1.37)	18.8	2.656	2.756	137	Immunology and microbiology: virology; Q1 Medicine: infectious diseases; Q1

^a^CS: CiteScore.

^b^SJR: SCImago Journal Rank.

^c^SNIP: Source-Normalized Impact per Paper.

^d^EMR: Eastern Mediterranean Region.

^e^The 2021 quarter corresponds to the SJR quartile rank per subject area, divided into 4 equal quartiles, with Q1 comprising the quarter of the journals with the highest values, Q2 the second-highest values, Q3 the third-highest values, and Q4 the lowest values.

### Publications by Subject Category

Each published document was categorized under 1 or more subject category in Scopus. Most of the retrieved documents (4211/6880, 61.21%) were categorized under medicine. Approximately 915 (13.30%) were categorized under social sciences and 627 (9.11%) under immunology and microbiology.

Of the 4211 documents categorized under medicine, 131 (3.11%) to 232 (5.51%) were labeled with index terms related to disease prevention, 120 (2.85%) to 262 (6.22%) with terms related to vaccination, 119 (2.83%) to 263 (6.25%) with terms related to treatment, 117 (2.78%) to 329 (7.81%) with mental health–related issues, 149 (3.34%) to 443 (10.52%) with disease severity terms, and 255 (6.56%) to 443 (10.52%) with mortality index terms (Table 3).

**Table 3:** Number of documents categorized under medicine and indexed with the index terms related to disease prevention, vaccination, treatment, mental health, disease severity, and mortality.

Index terms		Documents (N=4211), n (%)
**Disease prevention**		
	Quarantine	232 (5.51)
	Prevention and control	229 (5.44)
	Infection prevention	172 (4.08)
	Lockdown	167 (3.97)
	Social distancing	149 (3.54)
	Handwashing	131 (3.11)
**Vaccination**		
	Vaccination	262 (6.22)
	SARS-CoV-2 vaccine	156 (3.70)
	Vaccine	120 (2.85)
**Treatment**		
	Hydroxychloroquine	263 (6.25)
	Lopinavir	160 (3.80)
	Azithromycin	146 (3.47)
	Treatment outcome	122 (2.90)
	Antivirus agent	119 (2.83)
**Mental health**		
	Anxiety	329 (7.81)
	Psychology	272 (6.46)
	Depression	266 (6.32)
	Mental health	259 (6.15)
	Mental stress	121 (2.87)
	Stress	117 (2.78)
**Disease severity**		
	Disease severity	443 (10.52)
	Hospitalization	383 (9.10)
	Intensive care unit	327 (7.77)
	Hospital admission	249 (5.91)
	Length of stay	149 (3.54)
**Mortality**		
	Mortality	443 (10.52)
	Mortality rate	255 (6.56)

### Citation Metrics

The retrieved documents received a total of 53,516 citations, with an average of 7.78 (SD 34.30) citations per document (median 1, range 0-1462). The number of documents receiving ≥10, ≥50, ≥100, or ≥500 citations was 1112, 186, 78, and 5, respectively. Around 2758 (40.09%) of the 6880 publications had 0 citations.

The 3 most cited documents were all open-access multicountry collaborations, with the citation frequency equivalent to the 99th citation benchmarking percentile. The most cited document was “Physical Distancing, Face Masks, and Eye Protection to Prevent Person-to-Person Transmission of SARS-CoV-2 and COVID-19: A Systematic Review and Meta-Analysis,” which was a collaborative work between the American University of Beirut in Lebanon and researchers from Ontario, Canada, funded by WHO and published in the *Lancet* in 2020. It received a total of 1462 citations (n=1459, 99.8%, excluding author self-citations).

The second-most cited article was “The Fear of COVID-19 Scale: Development and Initial Validation,” which was published in 2020 in the *International Journal of Mental Health and Addiction* and was a collaboration between 3 universities in Iran (Tabriz University of Medical Sciences, Qazvin University of Medical Sciences, and Baqiyatallah University of Medical Sciences) and Nottingham Trent University in the United Kingdom, Jönköping University in Sweden, and the Hong Kong Polytechnic University. It received a total of 1174 citations (n=1092, 93.01%, excluding self-citation by authors).

The third-most cited publication was “The SARS, MERS and Novel Coronavirus (COVID-19) Epidemics, the Newest and Biggest Global Health Threats: What Lessons Have We Learned?,” which was published in the *International Journal of Epidemiology*. From the EMR, the National University of Medical Sciences in Pakistan and the Ofogh Kourosh Chain Stores in Iran collaborated with several other countries. This publication was funded by the National Key Research and Development Program of China. This study received a total of 664 citations (n=661, 99.54%, excluding self-citation by authors) and was published in 2021.

The top 5 most published authors, each publishing more than 25 documents on the topic (range 25-37), were from KSA (2 authors), Qatar (2 authors), and Jordan (1 author). Two of the authors, one from KSA and another from Qatar, were associated with their respective Ministry of Health, while the rest were associated with universities or medical centers in the region. Their average H-index on Scopus was 60 (SD 34; median 49, range 12-96), and their median citation frequency reported on Scopus was 10,094 (range 421-93,585).

### Visualization of Similarities and Associations

#### Coauthorship

**Figure 2:**
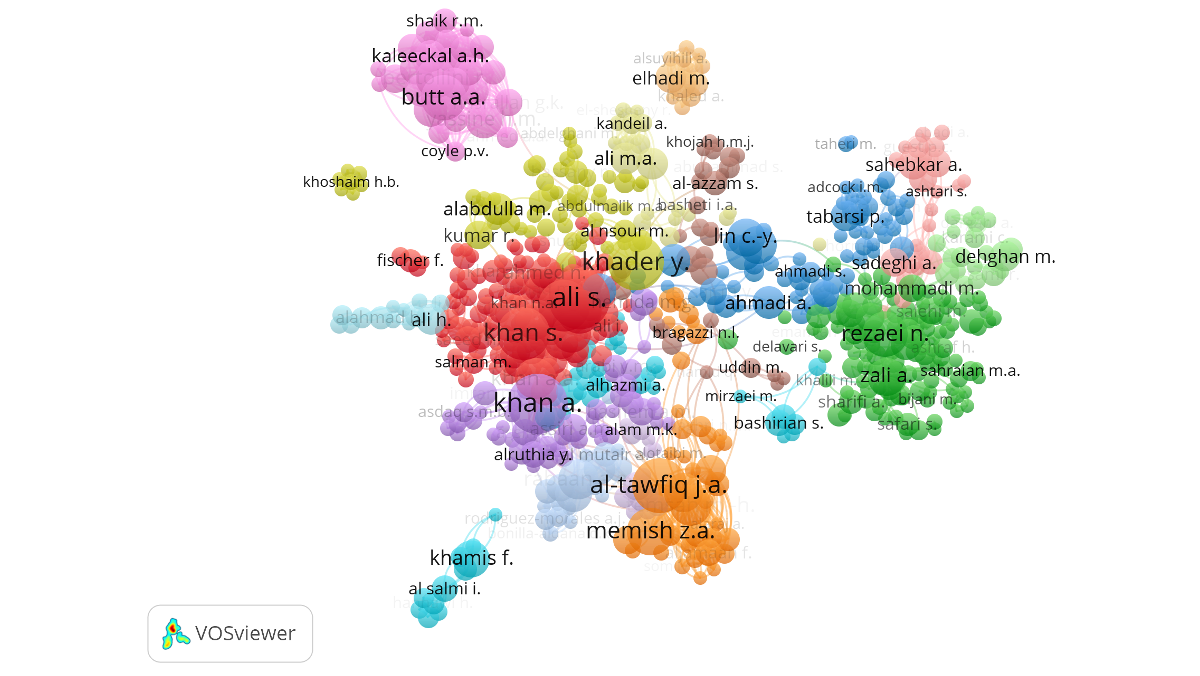
VOSviewer network of author coauthorship map representing 16 clusters of collaborations on COVID-19 research in the EMR. Included authors (N=719) were those with at least 5 publications, with up to 25 authors per publication, and who had been cited at least 5 times. EMR: Eastern Mediterranean Region; VOSviewer: Visualization of Similarities Viewer.

**Figure 3:**
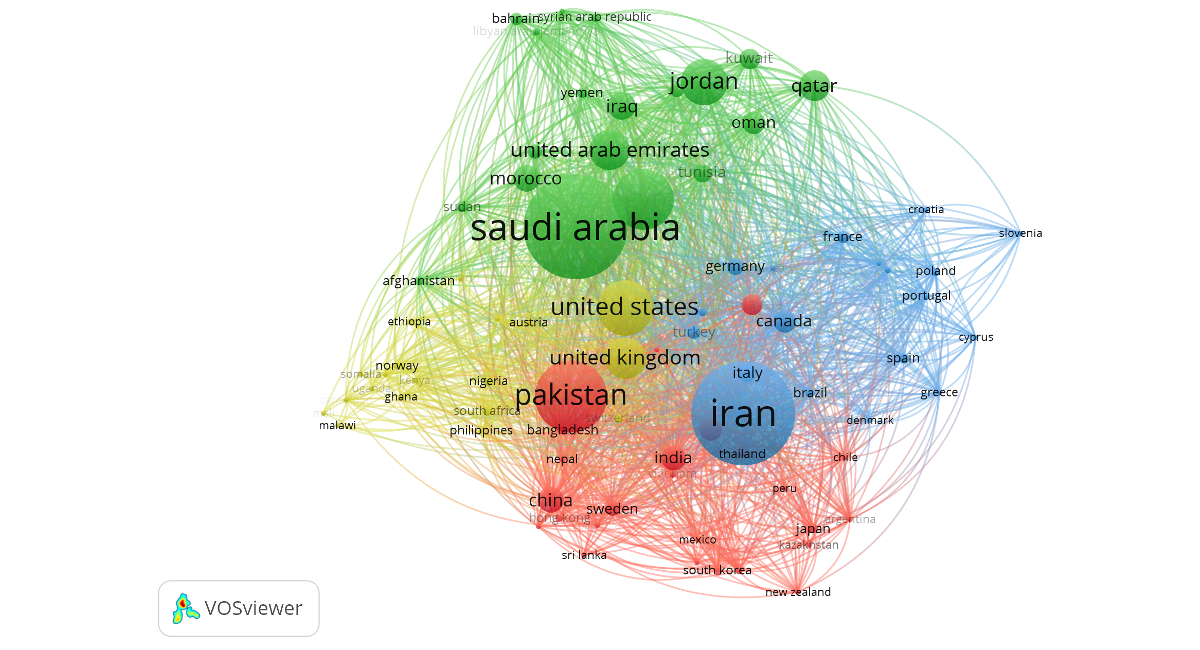
VOSviewer network of country coauthorship map weighed by the number of documents and representing 4 clusters of collaborations on COVID-19 research in the EMR. Included authors (N=82) were those with at least 5 publications, with up to 25 countries collaborating per publication. EMR: Eastern Mediterranean Region; VOSviewer: Visualization of Similarities Viewer.

A total of 26,798 authors were identified to have worked on publications with ≤25 authors. Of those, 796 (2.97%) met the threshold of having published ≥5 papers on the topic and being cited ≥5 times. Of the 796, 719 (90.33%) authors were connected in a total of 16 different clusters (Figure 2). Similarly, 285 countries were identified to have coauthored publications (with ≤25 countries per publication). Of those, 82 (28.77%) countries had ≥5 papers published on the topic and were connected in 4 clusters (Figure 3). In both figures, the size of the circles represents the number of documents published by the author or country, and the thickness of the lines depicts the size of the collaboration between the authors or countries (Figures 2 and 3, respectively).

**Figure 4:**
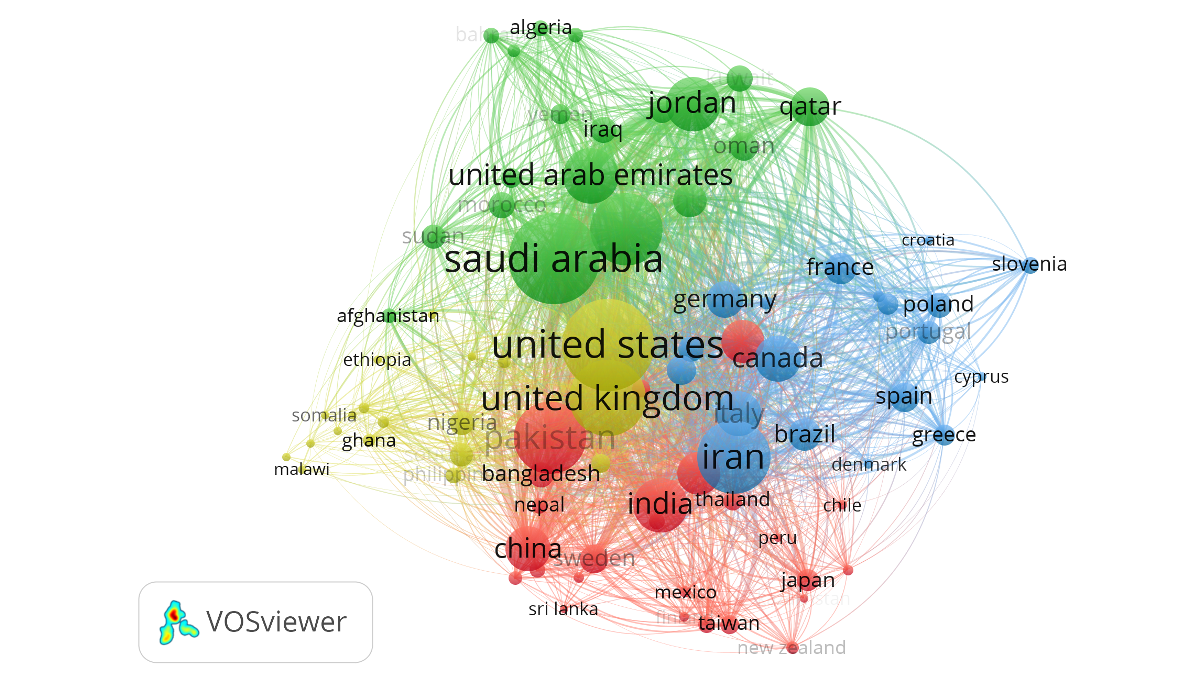
VOSviewer network of country coauthorship map, weighted by the total link strength, representing 4 clusters of collaborations on COVID-19 research in the EMR. Included countries (N=82) were those with at least 5 publications, with up to 25 countries collaborating per publication. EMR: Eastern Mediterranean Region; VOSviewer: Visualization of Similarities Viewer.

With regard to connections and collaborations, the EMR country with the most connections to other countries was Iran, with 77 links and a total link strength of 1279. KSA and Egypt came second, with 76 links each and a total link strength of 1759 and 1251, respectively. Pakistan had 75 links and a total link strength of 1247, the UAE had 68 links with a total link strength of 833, and Jordan had 66 links with a total link strength of 815. The United States and the United Kingdom had the most collaboration links (81 and 78, respectively), with a link strength of 1776 and 1361, respectively (Figure 4).

#### Co-occurrence

**Figure 5:**
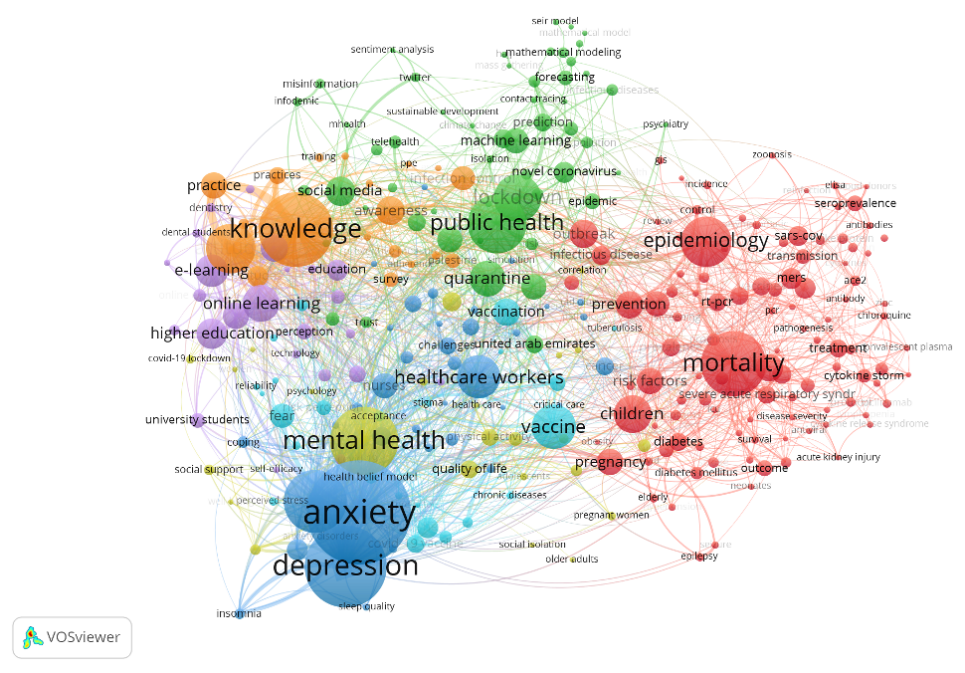
VOSviewer network of author keyword co-occurrence map weighted by occurrence, representing 7 clusters of keywords relating to COVID-19 research in the EMR. Included keywords (N=290) were those occurring at least 10 times. EMR: Eastern Mediterranean Region; VOSviewer: Visualization of Similarities Viewer.

**Figure 6:**
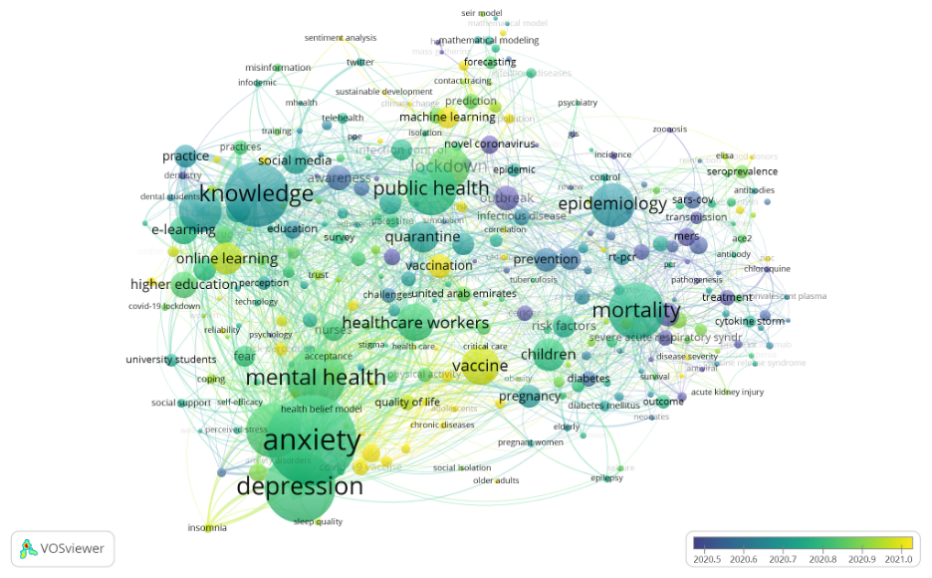
VOSviewer overlay visualization of author keyword relating to COVID-10 research in the EMR, weighted by occurrence and scored by the average publications per year. Included keywords (N=290) were those occurring at least 10 times. EMR: Eastern Mediterranean Region; VOSviewer: Visualization of Similarities Viewer.

There were 290 high-frequency keywords, occurring ≥10 times, linked in 7 distinct clusters (Figure 5). The cluster containing the most connected keywords was related to epidemiology and mortality. The second cluster included anxiety and depression, which were the two most occurring keywords, occurring 260 and 198 times, respectively (Table 4). The most recent themes included vaccines, vaccination, machine learning, and online learning (Figure 6).

**Table 4:** The 15 most occurring author keywords, with their frequency of occurrence, total link strength, and cluster number to which they belong.

Author keyword	Frequency of occurrence	Total link strength	Cluster number
Anxiety	260	573	1
Depression	198	454	1
Knowledge	178	406	2
Mental health	160	288	5
Mortality	153	186	3
Stress	147	346	6
Public health	135	163	4
Epidemiology	115	140	3
Attitude	111	246	2
Lockdown	107	126	4
Vaccine	98	128	6
Health care workers	95	151	1
Children	84	76	3
Online learning	80	73	7
Quarantine	78	120	4

## Discussion

### Principal Findings

The COVID-19 pandemic has led to unprecedented global research activity. To the best of our knowledge, our bibliometric analysis is the first to quantify the published literature on COVID-19 research that has been conducted by countries in the EMR up until early 2022. We provided a descriptive evidence-based analysis of the published research, identifying leading countries and organizational affiliations, with visualizations of collaborations and evolution of COVID-19 research output. Research on COVID-19 in the region has been led by authors and institutions from Iran and KSA. There were multiple regional collaborative efforts; however, international collaboration is limited. Output focuses on COVID-19 epidemiology, mortality, and anxiety and depression. Recently, interest has been shifting more toward topics related to vaccination, machine learning, and online learning.

Our results show that KSA and Iran are leading COVID-19 research in the EMR in terms of the number of publications. These results are not surprising, as KSA also leads the COVID-19 publications in the Arab world, with a 35.65% share of research production [33], and Iran has been the largest contributor to biomedical and health research in the EMR, with a 39% share during 2004-2013 [34].

The high number of COVID-19 research publications in both countries, especially by their leading universities (Tehran University of Medical Sciences in Iran and King Saud University in KSA), is due to many factors. First, both countries have relatively large populations, 35 million and 84 million in 2020, respectively [35], and both were among the countries most affected by the pandemic in the region, especially Iran [8]. Since the beginning of the pandemic and until April 1, 2022, more than 7 million confirmed COVID-19 cases were reported in Iran [8], which is the highest number in the region. The rapid spread of the disease in Iran in several waves was associated with many unique political, social, cultural, economic, and religious dimensions [36], providing a stimulating environment for research activities. For example, religious tourism in Qom, a pilgrimage site in Iran, which continued during the pandemic, was a major source of spread to other Iranian cities, Pakistan, and KSA in the early stages of the pandemic [37-39]. In KSA, mass gathering events, such as Umrah and Hajj, which were put on hold during the pandemic, may have also contributed to the high number of publications, not because of the high risk of transmission, but because of the successful measures that KSA took to prevent the progression of the COVID-19 pandemic locally and globally [39-41].

Second, both countries have invested extensively in the needed resources for medical and scientific research along the years. Iran has built a large human resource capacity with its unique Iranian Ministry of Health and Medical Education and its mandatory and credit-based continuing education programs for physicians for relicensing, along with the increasing number of Iranian journals indexed in international databases [42-44]. KSA has also rapidly become a major player in scientific research in the EMR, as research and development (R&D) is 1 of the main pillars of the Saudi Vision 2030, with the specific goal of having at least 5 Saudi universities among the top 200 in the world by 2030 [45,46]. These reasons may also explain why countries with fragile health systems, war torn, or with limited resources, such as Afghanistan, Djibouti, Somalia, Libya, and Syria, were less research-productive, although the number of cases in these countries may be higher than that officially reported and mandates further investigations.

Nevertheless, neither Iran nor KSA was reported to be at the top of the global COVID-19 literature in a recent global bibliometric analysis [47]. This adds to previous findings showing that the EMR share in global health research publications is low [34]. It is worth noting that Iran and KSA, like many other countries, faced various economic challenges during the pandemic that may have affected their research capacities. KSA was severely affected economically due to the radical decline in the global demand for oil, in parallel with the global economic recession, which was reflected in the sharp decline in oil prices during the first and second waves of the pandemic [48]. COVID-19 has also severely affected the Iranian economy, exacerbated by the concomitant international sanctions [49]. Generally, evidence shows that more support is needed for biomedical and health research in many of the EMR countries and institutions, especially those identified in our analysis as least productive.

International collaboration in COVID-19 research appears to be low in the EMR, as only 5.92% of the publications were associated with universities outside the EMR. The pandemic has shown that gaps in global cooperation, whether in research and information sharing, vaccine development and deployment, or travel policy, have affected the speed and equity of the global recovery [50]. In our study, the United States and the United Kingdom were at the center of collaboration and had the most extensive collaboration with EMR countries in COVID-19 research, similar to their role in COVID-19 research in the Arab world [33]. This could be due to the fact that both countries are among the top countries in scientific research [51] and specifically in global COVID-19 research [47]. The cross-border international flow of knowledge in the form cross-affiliation among researchers in the EMR with different international entities can be explained in several ways. First, the United States and the United Kingdom were by far the largest health donors to global health in 2019, with US $8.1 billion and US $2.9 billion, respectively [52], and when it comes to a donor-funded research project, most research funders require publication as a condition of a grant [53], triggering a cascade of cross-border research publications. Second, the United Kingdom and the United States have a network of research-intensive universities and scholarships, and both countries are hubs for international students, including those from the EMR, which could encourage cross-border collaboration. For example, the number of KSA students in the United Kingdom ranked eighth among international students from non–European Union countries, according to Higher Education Statistics Agency statistics for 2020/2021 [54]. In the United States, the number of KSA students ranked fourth among international students for the 2019-2020 academic year [55].

Most of the identified publications did not report a funding source, which could be due to limited funding opportunities or simply the likely dominance of cross-sectional studies and reviews, which are relatively less expensive, take less time to conduct, and require less infrastructure than, for example, clinical trials or prospective cohort studies [56]. The limited funding opportunities in the EMR may be related to the chronic underinvestment in research and development systems in the region, both financially and politically [57], exacerbated by the economic damage caused by the COVID-19 pandemic and the overreliance of some regional countries on external funding [58], although some major donors, such as the United Kingdom, have recently reduced their external funding [59]. Studies have also shown that research in the health care sector is not a top funding priority for governments, the private sector, or organizations in the EMR [57] and that R&D spending as a percentage of the gross domestic product (GDP) in the region is among the lowest in the world, despite significant differences in the GDP among member countries [58,60,61]. In EMR countries, R&D spending as a percentage of the GDP ranged from as low as 0.02% in Syria and Sudan in 2015 and 2019, respectively, to 1.3% in the UAE in 2018 compared to the global average of 2.27% in 2018 [58,61].

In our analysis of institutions funding COVID-19 research in the EMR, we found that 3 of the top 4 funders are from KSA, which is not surprising, given that KSA’s spending on R&D as a percentage of the GDP was 0.82 in 2013 [61] and is likely to increase, given that KSA’s GDP is still high [60], and the 2030 vision for R&D to increase universities’ competitiveness and ranking will continue to create a more favorable environment for research [45].

Network analysis of coauthorship showed that the COVID-19 flow of information between EMR authors comes from a large and highly interconnected network with few isolated and small clusters. This indicates a high level of regional collaboration, either because of prepandemic collaboration and networking or because of governments and organizations’ role in fostering collaboration since the onset of the pandemic. Regarding linkages, Iran had the most connections, consistent with another study showing that Iranian researchers have well-established international collaborations with leading countries in COVID-19 research [49].

Cultural differences could possibly have shaped COVID-19 research collaboration in EMR countries, which can be explained by Hofstede's theory of cultural dimensions [62]. A recent study analyzing research collaboration in the area of business and economics by 11 countries, including Iran from the EMR, before and after the onset of the pandemic, showed that Hofstede's uncertainty avoidance domain significantly influences the characteristics of research collaboration networks [63]. Uncertainty avoidance explains how cultures adjust to change and cope with uncertainty [62], and the pandemic has caused major changes and uncertainties at all levels of society worldwide [64]. However, using cross-national data from different EMR countries and interpreting them in the context of Hofstede’s model of cultural dimensions will provide more accurate results and explore more details of the impact of culture on COVID-19 research collaboration in the EMR.

Evaluation of the co-occurrence network for keywords showed that the EMR COVID-19 research covers a wide range of COVID-19–related areas. The most prevalent keywords were anxiety and depression, which may be related to the global increase in both conditions during the pandemic [65,66], especially in the EMR, given the limited mental health services and widespread stigma [67]. In addition, the EMR is fertile ground for high exposure to misleading and unknown information, which may exacerbate the status of mental disorders [68]. Recently developing topics in EMR COVID-19 research include vaccines, immunization, machine learning, and online learning. However, future research needs to address other underresearched topics, such as ethics, infodemiology, and the human-animal interface, in line with EMR-specific challenges and the recent recommendation of the WHO Research and Innovation Report for COVID-19 [69-71].

### Limitations

Our study was limited by factors that are inherent to its bibliometric nature, including the lack of ability to assess the quality of published documents; the fact that the number of citations may be misleading; and authors, countries, or institutions publishing early in 2020 are more likely to be cited than authors, countries, or institutions publishing in 2022. Additionally, within the EMR, a number of documents are published in Arabic, or other local languages, and in local journals and thus might not be indexed by Scopus and so would have been missed by this analysis. As is the case with other bibliometric studies, the results are dependent on the indexing of the databases, and even though Scopus was used for this bibliometric study, it should not be considered a comprehensive source of all published literature.

### Conclusion

The majority of available research on COVID-19 in the EMR is published by authors and institutions from Iran and KSA, with a paucity of publications from other EMR countries. Studies from these other countries are crucial to identify context-specific public health challenges and determine culturally and socially appropriate interventions and best buys. There were multiple regional collaborative efforts; however, international collaboration was limited, even though a pandemic is an opportune time for international communities to join forces. Recently, and in line with global interest, regional interest has been shifting more toward topics related to vaccination, machine learning, and online learning. This gradual shift to relatively new concepts can be considered a major step in the forward move of the state of knowledge of the EMR. Understanding the current state of research is instrumental to future research production, and our study will inform regional research initiatives on emerging concepts, as well as opportunities for collaboration and funding.

## Appendix

Multimedia Appendix 1 Scopus search strategy.

## Abbreviations

CS: CiteScore
EMR: Eastern Mediterranean Region
GDP: gross domestic product
KSA: Kingdom of Saudi Arabia
R&D: research and development
SJR: SCImago Journal Rank
SNIP: Source-Normalized Impact per Paper
UAE: United Arab Emirates
VOSviewer: Visualization of Similarities Viewer
WHO: World Health Organization

## References

[1] Telenti A, Arvin A, Corey L, Corti D, Diamond MS, García-Sastre A, Garry RF, Holmes EC, Pang PS, Virgin HW (2021). After the pandemic: perspectives on the future trajectory of COVID-19. Nature.

[2] Sidik SM (2022). Vaccines protect against infection from Omicron subvariant - but not for long. Nature.

[3] Ye Y, Zhang Q, Wei X, Cao Z, Yuan H, Zeng DD (2022). Equitable access to COVID-19 vaccines makes a life-saving difference to all countries. Nat Hum Behav.

[4] Lami F, Elfadul M, Rashak H, Al Nsour M, Akhtar H, Khader Y, Hussein AM, Naciri M, Samy S, Ghaleb Y, Taha H, Hussein A, Ali NA, Hussein R, Ikram A, Rahman FU, Khan MM, Adam R, Ahmed AY, Afifi S (2022). Risk factors of COVID-19 critical outcomes in the Eastern Mediterranean Region: multicountry retrospective study. JMIR Public Health Surveill.

[5] World Health Organization The COVID-19 Pandemic in the Eastern Mediterranean Region.

[6] Nabeth P, Hassan M, Adib K, Abubakar A, Brennan R (2021). Addressing the real trajectory of COVID-19 in the Eastern Mediterranean region – authors' reply. Lancet.

[7] Alsubaie S, Alshahrani H, Alshahrani A, Asiri A, Alfaifi A, Ibrahim RA, Alqahtani W (2021). Denial attitude towards COVID-19 among general population in Saudi Arabia. Eur Psychiatr.

[8] World Health Organization Coronavirus (COVID-19) Dashboard With Vaccination Data.

[9] World Health Organization Tackling Infectious Disease Outbreaks in the Eastern Mediterranean Region.

[10] Salameh P (2020). COVID-19 in the Eastern Mediterranean Region: testing frequency, cumulative cases and mortality analysis. East Mediterr Health J.

[11] ReliefWeb Many Countries in the Eastern Mediterranean Region Lagging behind in Vaccination Efforts.

[12] Raynaud M, Goutaudier V, Louis K, Al-Awadhi S, Dubourg Q, Truchot A, Brousse R, Saleh N, Giarraputo A, Debiais C, Demir Z, Certain A, Tacafred F, Cortes-Garcia E, Yanes S, Dagobert J, Naser S, Robin B, Jouven X, Reese PP, Loupy A, Bailly (2021). Impact of the COVID-19 pandemic on publication dynamics and non-COVID-19 research production. BMC Med Res Methodol.

[13] Palayew A, Norgaard O, Safreed-Harmon K, Andersen TH, Rasmussen LN, Lazarus JV (2020). Pandemic publishing poses a new COVID-19 challenge. Nat Hum Behav.

[14] Cunningham E, Smyth B, Greene D (2021). Correction: Collaboration in the time of COVID: a scientometric analysis of multidisciplinary SARS-CoV-2 research. Humanit Soc Sci Commun.

[15] Mol A, Hardon A (2020). What COVID-19 may teach us about interdisciplinarity. BMJ Glob Health.

[16] Sigala M, Utkarsh (2021). A bibliometric review of research on COVID-19 and tourism: reflections for moving forward. Tour Manag Perspect.

[17] Okolie CC, Ogundeji AA (2022). Effect of COVID-19 on agricultural production and food security: a scientometric analysis. Humanit Soc Sci Commun.

[18] Brika SKM, Chergui K, Algamdi A, Musa AA, Zouaghi R (2021). E-learning research trends in higher education in light of COVID-19: a bibliometric analysis. Front Psychol.

[19] Verma S, Gustafsson A (2020). Investigating the emerging COVID-19 research trends in the field of business and management: a bibliometric analysis approach. J Bus Res.

[20] DEVEX COVID Funding Visualisation.

[21] Diptyanusa A, Zablon KN (2020). Addressing budget reduction and reallocation on health-related resources during COVID-19 pandemic in malaria-endemic countries. Malar J.

[22] Oladele TT, Olakunde BO, Oladele EA, Ogbuoji O, Yamey G (2020). The impact of COVID-19 on HIV financing in Nigeria: a call for proactive measures. BMJ Glob Health.

[23] World Health Organization The Work of WHO in the Eastern Mediterranean Region: Annual Report of the Regional Director 2019.

[24] Qatar Foundation QF Fast-Track Research Funding Program Boosts Fight Against COVID-19.

[25] Falagas ME, Pitsouni EI, Malietzis GA, Pappas G (2008). Comparison of PubMed, Scopus, Web of Science, and Google Scholar: strengths and weaknesses. FASEB J.

[26] Scopus: Content Coverage Guide.

[27] World Health Organaization WHO EMRO Countries.

[28] Roldan-Valadez E, Salazar-Ruiz SY, Ibarra-Contreras R, Rios C (2019). Current concepts on bibliometrics: a brief review about impact factor, eigenfactor score, CiteScore, SCImago Journal Rank, Source-Normalised Impact per Paper, H-index, and alternative metrics. Ir J Med Sci.

[29] Elsevier Scopus Blog Journal Metrics in Scopus: Source Normalized Impact per Paper (SNIP).

[30] Centers for Disease Control and Prevention Scimago Journal and Country Rank.

[31] Centre for Science and Technology Studies, Leiden University Welcome to VOSviewer.

[32] Jan VEN, Waltman L VOSviewer Manual: Manual for VOSviewer version 1.6.16.

[33] Zyoud SH (2021). The Arab region's contribution to global COVID-19 research: bibliometric and visualization analysis. Global Health.

[34] Tadmouri GO, Mandil A, Rashidian A (2019). Biomedical and health research geography in the Eastern Mediterranean Region. East Mediterr Health J.

[35] World Bank World Population.

[36] Khankeh H, Farrokhi M, Roudini J, Pourvakhshoori N, Ahmadi S, Abbasabadi-Arab M, Bajerge NM, Farzinnia B, Kolivand P, Delshad V, Khanjani MS, Ahmadi-Mazhin S, Sadeghi-Moghaddam A, Bahrampouri S, Sack U, Stueck M, Domres B (2021). Challenges to manage pandemic of coronavirus disease (COVID-19) in Iran with a special situation: a qualitative multi-method study. BMC Public Health.

[37] Badshah SL, Ullah A, Badshah SH, Ahmad I (2020). Spread of novel coronavirus by returning pilgrims from Iran to Pakistan. J Travel Med.

[38] Ghafari M, Hejazi B, Karshenas A, Dascalu S, Kadvidar A, Khosravi MA, Abbasalipour M, Heydari M, Zeinali S, Ferretti L, Ledda A, Katzourakis A (2021). Lessons for preparedness and reasons for concern from the early COVID-19 epidemic in Iran. Epidemics.

[39] Hoang V, Gautret P, Memish ZA, Al-Tawfiq JA (2020). Hajj and Umrah mass gatherings and COVID-19 infection. Curr Trop Med Rep.

[40] Basahel S, Alsabban A, Yamin M (2021). Hajj and Umrah management during COVID-19. Int J Inf Technol.

[41] Alshammari SM, Almutiry WK, Gwalani H, Algarni SM, Saeedi K (2021). Measuring the impact of suspending Umrah, a global mass gathering in Saudi Arabia on the COVID-19 pandemic. Comput Math Organ Theory.

[42] Mansoori P (2018). Evolution of Iran's health research system over the past 50 years: a narrative review. J Glob Health.

[43] Faghihi SA, Khankeh HR, Hosseini SJ, Soltani Arabshahi SK, Faghih Z, Shirazi M (2017). Impractical CME programs: influential parameters in Iran. Med J Islam Repub Iran.

[44] Chen L, Evans T, Anand S, Boufford JI, Brown H, Chowdhury M, Cueto M, Dare L, Dussault G, Elzinga G, Fee E, Habte D, Hanvoravongchai P, Jacobs M, Kurowski C, Michael S, Pablos-Mendez A, Sewankambo N, Solimano G, Stilwell B, de Waal A, Wibulpolprasert S (2004). Human resources for health: overcoming the crisis. Lancet.

[45] Ul Haq I, Ur Rehman S, Al-Kadri HM, Farooq RK (2020). Research productivity in the health sciences in Saudi Arabia: 2008-2017. Ann Saudi Med.

[46] Saudi Ministry of Education R&D in the Kingdom.

[47] Wang P, Tian D (2021). Bibliometric analysis of global scientific research on COVID-19. J Biosaf Biosecur.

[48] Alharbi R (2021). Impact of COVID-19 on Saudi Arabia's economy: evidence from macro-micro modelling. PSS Res Rev.

[49] Shamsi A, Mansourzadeh MJ, Ghazbani A, Khalagi K, Fahimfar N, Ostovar A (2020). Contribution of Iran in COVID-19 studies: a bibliometrics analysis. J Diabetes Metab Disord.

[50] Jit M, Ananthakrishnan A, McKee M, Wouters OJ, Beutels P, Teerawattananon Y (2021). Multi-country collaboration in responding to global infectious disease threats: lessons for Europe from the COVID-19 pandemic. Lancet Reg Health Eur.

[51] Nature Index 2021 Tables: Countries/Territories.

[52] Donor Tracker Global Health.

[53] Buckley RC (2022). Stakeholder controls and conflicts in research funding and publication. PLoS One.

[54] Study in the UK International Student Statistics in UK 2022.

[55] Migration Policy Institute International Students in the United States.

[56] Parab S, Bhalerao S (2010). Study designs. Int J Ayurveda Res.

[57] Ismail SA, McDonald A, Dubois E, Aljohani FG, Coutts AP, Majeed A, Rawaf S (2013). Assessing the state of health research in the Eastern Mediterranean Region. J R Soc Med.

[58] El Rabbat M, El-Jardali F, Fadlallah R, Soror S, Ahmadnezhad E, Badr E, Dabis J (2021). Funding for health policy and systems research in the Eastern Mediterranean region: amount, source and key determinants. Public Health Res Pract.

[59] Bird V, He FJ, Heritage P, Kelly P, MacGregor G, Martineau A, McCoy D, Montag D, Prendergast AJ, Priebe S, Russo G, van Loggerenberg F (2021). The United Kingdom's global health funding cuts will exacerbate inequities. Nat Microbiol.

[60] Worldometer GDP by Country.

[61] World Bank Research and Development Expenditure (% of GDP).

[62] Hofstede G (2011). Dimensionalizing cultures: the Hofstede model in context. ORPC.

[63] Damaševičius R, Zailskaitė-Jakštė L (2022). Impact of COVID-19 pandemic on researcher collaboration in business and economics areas on national level: a scientometric analysis. J Doc.

[64] Romiti GF, Talerico G (2021). Embracing the uncertainty: an important lesson from COVID-19. J Gen Intern Med.

[65] Su Z, McDonnell D, Wen J, Kozak M, Abbas J, Šegalo S, Li X, Ahmad J, Cheshmehzangi A, Cai Y, Yang L, Xiang Y (2021). Mental health consequences of COVID-19 media coverage: the need for effective crisis communication practices. Glob Health.

[66] Nochaiwong S, Ruengorn C, Thavorn K, Hutton B, Awiphan R, Phosuya C, Ruanta Y, Wongpakaran N, Wongpakaran T (2021). Global prevalence of mental health issues among the general population during the coronavirus disease-2019 pandemic: a systematic review and meta-analysis. Sci Rep.

[67] Charara R, Forouzanfar M, Naghavi M, Moradi-Lakeh M, Afshin A, Vos T, Daoud F, Wang H, El Bcheraoui C, Khalil I, Hamadeh RR, Khosravi A, Rahimi-Movaghar V, Khader Y, Al-Hamad N, Makhlouf Obermeyer C, Rafay A, Asghar R, Rana SM, Shaheen A, Abu-Rmeileh NME, Husseini A, Abu-Raddad LJ, Khoja T, Al Rayess ZA, AlBuhairan FS, Hsairi M, Alomari MA, Ali R, Roshandel G, Terkawi AS, Hamidi S, Refaat AH, Westerman R, Kiadaliri AA, Akanda AS, Ali SD, Bacha U, Badawi A, Bazargan-Hejazi S, Faghmous IAD, Fereshtehnejad S, Fischer F, Jonas JB, Kuate Defo B, Mehari A, Omer SB, Pourmalek F, Uthman OA, Mokdad AA, Maalouf FT, Abd-Allah F, Akseer N, Arya D, Borschmann R, Brazinova A, Brugha TS, Catalá-López F, Degenhardt L, Ferrari A, Haro JM, Horino M, Hornberger JC, Huang H, Kieling C, Kim D, Kim Y, Knudsen AK, Mitchell PB, Patton G, Sagar R, Satpathy M, Savuon K, Seedat S, Shiue I, Skogen JC, Stein DJ, Tabb KM, Whiteford HA, Yip P, Yonemoto N, Murray CJL, Mokdad AH (2017). The burden of mental disorders in the Eastern Mediterranean Region, 1990-2013. PLoS One.

[68] IFEX MENA Region Battles the Infodemic: From Fake News to Hashtag-Washing in the Region’s Ongoing Information Wars.

[69] World Health Organization COVID-19 Research and Innovation Powering the World’s Pandemic Response: Now and in the Future.

[70] Neff EP (2021). Keeping an eye on the human-animal interface. Lab Anim (NY).

[71] Singla R, Mishra A, Joshi R, Jha S, Sharma AR, Upadhyay S, Sarma P, Prakash A, Medhi B (2020). Human animal interface of SARS-CoV-2 (COVID-19) transmission: a critical appraisal of scientific evidence. Vet Res Commun.

