# Supplementary material for: Published Research on COVID-19 in the Eastern Mediterranean Region: Bibliometric Analysis
Source: Interact J Med Res. 2022 Jul 19;11(2):e38935. doi: 10.2196/38935 (PMC9384956; doi:10.2196/38935)
Supplement: Multimedia Appendix 1 [file ijmr_v11i2e38935_app1.doc]

**Scopus Search Strategy**

**Concept 1: Eastern Mediterranean Regio Countries**

#1. ( TITLE-ABS-KEY ( afghani*  OR  bahrain*  OR  egypt*  OR  iran*  OR  iraq*  OR  jordan*  OR

kuwait*  OR  liban*  OR  libya*  OR  morocc*  OR  oman*  OR  pakistan*  OR  palestin*  OR  qatar*  OR  saudi*  OR  ksa  OR  somali*  OR  sudan*  OR  syria*  OR  tunisia*  OR  yemen*  OR  emirat*  OR  uae OR ( "eastern mediterranean region" )  OR  ( "middle east*" )  OR  ( "north* africa" )  OR  ( "mena region" )  OR  emr )

**Concept 2: COVID-19**

#2. TITLE-ABS-KEY ( "COVID-19"  OR "COVID 19" OR “COVID19” OR "COVID 2019" OR "severe acute respiratory syndrome coronavirus 2" OR  "SARS-CoV-2" OR “SARS CoV 2” OR “SARS CoV2” OR “SARS-CoV2” OR “sars2” OR “sars-2” OR "2019-nCoV" OR "2019 ncov" OR ( ( novel OR new OR 2019 OR wuhan OR hubei OR china ) AND ( coronavirus OR covid ) ) )

**Limits:** Year > 2018, English language, and EMR affiliated countries

#3. ( PUBYEAR  >  2018 )

#4. ( LANGUAGE ( "English" ) )

#5. ( DOCTYPE ( "ar"  OR  "re"  OR  "ch"  OR  "bk" ) )

#6. ( AFFILCOUNTRY ( ( "Saudi Arabia" )  OR  "Iran"  OR  "Pakistan"  OR  "Egypt"  OR  "Jordan"  OR  ( "United Arab Emirates" )  OR  "Qatar"  OR  "Iraq"  OR  "Morocco"  OR  "Oman"  OR  "Kuwait"  OR  "Tunisia"  OR  "Palestine"  OR  "Lebanon"  OR  "Bahrain"  OR  "Sudan"  OR  "Yemen"  OR  "Afghanistan"  OR  "Syrian Arab Republic"  OR  ( "Libyan Arab Jamahiriya" )  OR  "Somalia"  OR  "Djibouti" ) )

**Search:** #1 AND #2 AND #3 AND #4 AND #5 AND #6
